# Cerebro-Spinal Fever (Epidemic Cerebro-Spinal Meningitis)

**Published:** 1907-03

**Authors:** D. S. Davies, I. Walker Hall

**Affiliations:** Medical Officer of Health, Bristol; Professor of Pathology, University College, Bristol; Pathologist to the Bristol Royal Infirmary.


### CEREBRO-SPINAL FEVER (EPIDEMIC CEREBRO-SPINAL MENINGITIS).

BY

D. S. DAVIES, M.D.,
*Medical Officer of Health, Bristol,*

AND

I. WALKER HALL, M.D.,
*Professor of Pathology, University College, Bristol;*
*Pathologist to the Bristol Royal Infirmary.*

---

THE invasion of New York by cerebro-spinal fever in the winter and spring of 1904–5, when some 4,000 cases occurred,[1] and the more recent appearance of the disease in Glasgow (March, 1906), in Belfast, and some other districts, invite attention to its causation and prophylaxis.

It is generally recognised that a cerebro-spinal meningitis may be set up by various causes, such as disease of the middle ear, pneumococcal, tubercular, or streptococcal infection, or as a sequel to certain of the exanthemata; but during the last years, the form (cerebro-spinal fever) which exhibits marked though erratic infective properties under predisposing conditions at present ill-understood, has been found to be definitely associated with the organism of Weichselbaum, the diplococcus meningitidis intracellularis.

The *history* of the disease[2] is not without interest. Its occurrence is veiled before the nineteenth century, but in 1804–5 Vieusseaux, of Geneva, distinctly described it. In the following year it appeared in America (Massachusetts), and the United States have hardly been free from it since; it became widely spread during the Civil War. In Canada the outbreaks have been small, probably owing to the scattered population. In

[1] Flexner, *Brit. M. J.*, 1906, ii. 1023.

[2] Bruce Low, *Tr. Epidem. Soc. Lond.*, 1898–9, N.S., xviii. 53.

India it has been prevalent from time to time in the jails. Europe has never been quite free since the Geneva outbreak, in turn it spread to France, Italy and Denmark, and afterwards to nearly every European country. In France it was noticed that the outbreaks largely affected troops in garrison. A widespread outbreak occurred in Dantzig in 1865–6, when 1,900 were attacked, chiefly children.

The disease was first noted in Ireland in 1845–6, when it affected the Irish Constabulary and the workhouses at Dublin, Bray, and Belfast; again in 1866 it prevailed in the Dublin barracks and in the Curragh; in 1885–6–7 it again became epidemic in Ireland. In the first six months of 1900 there was an outbreak in Cork[1] involving 100 cases and 25 deaths.

In Scotland it has been shown that, though not generally recognised, the disease occurred from time to time in small outbreaks between 1877 and 1886.

In England and Wales also various groups of cases have from time to time been reported, but its nature appears to have not infrequently escaped recognition; it is probably not so rare as is generally supposed, and since 1890 there has apparently been a general tendency to ascribe obscure outbreaks to influenza.

*Local History.*—No widespread outbreak has been recorded in the Bristol district, but Stack[2] has recorded an interesting group of six cases received into the Royal Infirmary in 1900–1, in five of which the diagnosis was confirmed by lumbar puncture and recognition of the diplococcus intracellularis: the sixth case occurred in the same house as, and was the daughter of, a fatal case. He sums up the most constant conditions as: (1) An irregular temperature not assignable to other causes; (2) pain in the back of the head and neck; (3) general hyperæsthesia; (4) herpes labialis; (5) Kernig's sign (inability to extend the leg on the thigh when the thigh is at right angles to body); (6) discovery of specific diplococcus.

The following table, for which we are indebted to Dr. A. Rendle Short, gives a résumé of cases which have been treated in the Bristol Royal Infirmary since 1901:—

[1] *Brit. M. J.*, 1907, i. 461. [2] *Bristol M.-Chir. J.*, 1901, xix. 44.

| DATE OF ADMISSION. | SEX. | AGE. | SYMPTOMS, ETC. | RESULT. | ORGANISMS. | REMARKS. |
|---|---|---|---|---|---|---|
| 8.2.02 | F | 22 | Headache, optic neuritis, vomiting, squint, no fever | Cure.. | —— | Perhaps not proven |
| 2.6.02 | F | 14 | Headache, coma, hazy discs, herpes, no fever | Cure.. | —— | Doubtful |
| 27.4.03 | M | 37 | Coma, fever, delirium .. | Death | M. meningitidis (in nose also) | —— |
| 5.3.04 | M | 15 | Fever, delirium, headache retraction, herpes, hazy discs | Cure.. | M. meningitidis | —— |
| 26.5.04 | M | 15 | Fever, headache, herpes, Kernig's sign | Death | M. meningitidis | —— |
| 18.9.05 | M | 6/12 | Posterior basic meningitis, with retraction | Death | —— | Undoubtedly a form of C.S. meningitis |
| 31.12.05 | M | 33 | Headache, fever, coma, retraction | Death | —— | Proved at autopsy |
| 2.3.06 | F | 10 | Proptosis, sphenoidal sinusitis, basic meningitis | Death | M. meningitidis | —— |
| 22.2.06 | M | 3/12 | Posterior basic meningitis with retraction | Death | Diplococci and staphylococci | —— |

*Infectivity.*—The recorded facts in regard to cerebro-spinal fever are not so contradictory as they at first appear to be, and there is little doubt that the chief incidence of the disease is upon childhood, or upon young adult life,[1] and that in close communities, such as barracks or institutions, the disease is certainly, though apparently not very readily, communicable.

The seemingly sporadic habit of the disease, when introduced under conditions of less intimate association, as into a village, may be explained by the habits of the organism, which when grown artificially is readily attenuated, and may thus, if similar attenuation attend its natural distribution amongst individuals of varying resistance, give rise to ill-marked cases, or to "carrier" cases, by which the infection is preserved and handed on, exactly as in the case of diphtheria. Extensive investigations by Osterman showed that the meningococcus was present in the nasal cavities of 74 per cent. of the people who had been in the neighbourhood of the patients. Jehle points out that miners form specially good carriers, as the organism thrives best apart from light, and in a warm, moist atmosphere.

[1] Hirsch points out that the age limit in most epidemics is from 1 to 15, but in "military" epidemics the most vulnerable ages are from 18 to 24. —*Geographical and Historical Pathology*, vol. iii., New Sydenham Soc., 1886

It must not be forgotten that animals may also act as carriers. In the 1872 New York outbreak, and in the 1866 Irish outbreak, horses and pigs suffered badly from cerebro-spinal fever. Rabbits, mice and guinea-pigs are almost insusceptible to the action of the meningococcus.

The limits of an outbreak may, therefore, not necessarily be defined by the known cases, and, equally, the fatality of outbreaks is probably, as a rule, overstated.

This points to the need for early notification, before the specific organism has become "domesticated" in a district, so that confirmation of diagnosis by lumbar puncture and bacteriological examination may further the application of prompt preventive measures to the initial cases.

Farrar[1] insists with much cogency upon the infectivity of the disease, and adduces instances pointing to the conveyance of the infection by "fomites," by aerial infection during expectoration and sneezing, by kissing, and by the mediate agency of contacts. Its "limited" infectivity is, at the same time, indicated by its sometime presence in barracks without concurrent prevalence in the civil population, and by the converse condition.

The epidemiological facts point to the persistence of the organism with high potentiality of infection for long periods, followed by an explosive intensity upon occasion, as manifested in fatal "house-epidemics;" this activity is rapidly exhausted, and these localised outbreaks, intensely virulent within a narrow range, do not, as a rule, spread very far. In explanation of its explosive epidemic virulence, he hazards the suggestion of a symbiosis of the meningococcus with some other organism, such as the micrococcus catarrhalis, or Pfeiffer's bacillus.

*Prophylaxis.*—Two classes of people must be considered, namely the "infected" and the "infective." It is apparently necessary that the buccal and nasal cavities of possible "carriers" should be washed out night and morning with a good stream of warm dilute antiseptic solution, and swabs should be taken and submitted for bacteriological examination at regular intervals.

[1] Farrar, "The Infectivity of Cerebro-spinal Fever," *Tr. Epidem. Soc. Lond.*, 1905–6, N.S., vol. xxv. 245.

"Contacts" should be isolated and similarly treated.

As to the patient himself, effective isolation is very advisable. His nasal and buccal cavities and hands should be kept continuously clean. All the utensils he touches should be immediately disinfected. Handkerchiefs, towels, linen, bedclothes should be dipped at once after use into disinfectant solution. The room which the patient has occupied should be thoroughly disinfected

Mervyn Gordon recommends medical "Izal" for the purpose of buccal and nasal disinfection, and places potassium permanganate equal to mercury bichloride or silver nitrate. Almost any disinfectant will serve the purpose, provided it is used sufficiently strong.

Jehle employs pyozyanase, a proteolytic ferment. The introduction of five drops of this ferment serves to effectually kill the organism when present in the nasal fossæ.

*Diagnosis.*—Lumbar puncture should always be made in indefinite cases of meningitis. The skin should be specially well cleansed, and a needle with a small bore employed. The fluid should be received into a sterile tube, the tube at once plugged with sterile wool, and transmitted for chemical, cytological, and bacteriological examination. The characters of the cells afford useful information, and the coccus itself can be isolated within a few days.

The agglutination reaction is also of value. It may be produced by a sample of blood withdrawn from the patient on the sixth day and onwards.

The *general features* of the disease are thus described in the memorandum of the Local Government Board, issued in 1905:—

"An acute epidemic disease, characterised by profound disturbance of the cerebral nervous system, indicated at the outset chiefly by shivering, intense headache or vertigo, or both, and persistent vomiting; subsequently by delirium, often violent, alternating with somnolence or a shade of apathy or stupor; an acutely painful condition, with spasm—sometimes tetanoid—of certain groups of muscles, especially the posterior muscles of the neck, occasioning retraction of the head, and an increased sensitiveness of the surface of the body. Throughout the disease there is marked depression of the vital powers, not infrequently collapse, and in its course an eruption of vesicles, petechiæ, or purpuric spots, or mottling of the skin is apt to occur. If the disease tends to recovery, the symptoms gradually subside without any critical phenomena, and convalescence is protracted; if to a fatal termination, death is almost invariably preceded by coma."

Various forms are described, viz.:—

1. The fulminant form, attacking suddenly and killing quickly.
2. The simple form, having typical nervous symptoms.
3. The purpuric form, attended by hemorrhages.
4. The abortive form, with anomalous symptoms, running a short or irregular course.

A specific coccal pharyngitis or tonsilitis is a frequent early symptom.

The *predisposing causes* upon which chief importance has been placed are cold, dampness of soil, fatigue, and, in general, depressing influences; also the influence of insanitary conditions, especially such as generally accompany overcrowding.

*Treatment.*—In addition to the older remedies, withdrawal of fluid by lumbar puncture and the frequent use of hot baths are now generally advised. The proposed injection of 1 per cent. lysol into the spinal canal has not met with general application. Injections of mercurial cyanate and collargolsalbe may be placed in a similar category. Westenhœffer suggests incision of the atlanto-occipital ligament during the second week of the attack. This provides permanent drainage, prevents hydrocephalus, and permits of local applications being made.

*Serum Treatment.*—Many attempts have been made to obtain a curative serum. The earlier sera of Bonhoff, Lepierre, and Lingelsheim, obtained after injection of the dead and living cocci, had only slight protective effects. A similar result followed the earlier use of aggressin. The names of Wasserman, Kolle, Bruck, Bordet and Gengon, Mareschi, Neisser and Sachs, indicate the amount of work and the eminence of the workers upon this subject. The endeavour was next made to estimate the quantity and specificity of the immune bodies in the anti-serum. Inactivated specific serum—meningococcal serum—was placed in a test-tube, together with an extract from the cocci themselves, and with some normal guinea-pig serum, and kept at 56° C. for about half an hour. The amboceptors of the anti-serum, the receptors of the bacillary extract, and the complements of the fresh serum were brought thus together. The addition of an inactive hæmolytic system (serum and blood corpuscles) was now made in order to avoid ultimate hæmolytic action. After two hours in the incubator, and twenty-four hours on ice, specific amboceptors appear and anchor the complement. In this way the specificity and quantity of the amboceptors has been determined.

Five to ten c.c. of this serum are injected subcutaneously, and the injection is repeated once or twice on the following or alternate days.

Jochmann prepares a serum which is injected intra-spinally in quantities of 20–30 c.c., after removal of 20–50 c.c. of the spinal fluid by lumbar puncture. In this connection it may be cited that lumbar puncture itself, by the withdrawal of toxins and meningococci, is often followed by surprisingly good results.

The condition appears to be very favourably influenced by the use of the serum, but it is as yet too early to quote any definite statistics.

It is possible that "vaccine" treatment may play a useful part in later outbreaks.

*Pathological Anatomy.*—The main features consist of a thick, purulent, creamy exudate covering the base, and sometimes the cortex, of the brain. The Sylvian fissure is generally free from exudate, but the upper surface of the cerebellum is the seat of much exudate. These points are useful from a diagnostic standpoint. In long-standing cases the meninges are fibrous, and cerebral atrophy and serous, or purulent, internal hydrocephalus are frequent. The cord shows similar conditions.

In addition, meningo-encephalitis often occurs, the glandular organs generally show cloudy swelling, the spleen is enlarged, there is often purulent bronchitis and lobular pneumonia, pleurisy and pericarditis and joint troubles are not infrequent, and catarrh and punctiform hemorrhages are present throughout the alimentary tract, while the mesenteric glands are almost always enlarged.

The meningococcus can be obtained from either the nasal cavity or the pharynx. The organism appears to extend to the meninges by the lymphatics, although it is not possible to entirely exclude hæmatogenous paths of infection.

There are many gaps in our knowledge of this disease, and every case is worthy of minute and careful observation and record.

